# Hierarchical clustering of maximum parsimony reconciliations

**DOI:** 10.1186/s12859-019-3223-5

**Published:** 2019-11-27

**Authors:** Ross Mawhorter, Ran Libeskind-Hadas

**Affiliations:** Department of Computer Science, Harvey Mudd College, Claremont, California USA

**Keywords:** Phylogenetic trees, Maximum parsimony reconciliation, Duplication-transfer-loss model

## Abstract

**Background:**

Maximum parsimony reconciliation in the duplication-transfer-loss model is a widely-used method for analyzing the evolutionary histories of pairs of entities such as hosts and parasites, symbiont species, and species and genes. While efficient algorithms are known for finding maximum parsimony reconciliations, the number of such reconciliations can be exponential in the size of the trees. Since these reconciliations can differ substantially from one another, making inferences from any one reconciliation may lead to conclusions that are not supported, or may even be contradicted, by other maximum parsimony reconciliations. Therefore, there is a need to find small sets of best representative reconciliations when the space of solutions is large and diverse.

**Results:**

We provide a general framework for hierarchical clustering the space of maximum parsimony reconciliations. We demonstrate this framework for two specific linkage criteria, one that seeks to maximize the average support of the events found in the reconciliations in each cluster and the other that seeks to minimize the distance between reconciliations in each cluster. We analyze the asymptotic worst-case running times and provide experimental results that demonstrate the viability and utility of this approach.

**Conclusions:**

The hierarchical clustering algorithm method proposed here provides a new approach to find a set of representative reconciliations in the potentially vast and diverse space of maximum parsimony reconciliations.

## Background

Phylogenetic tree reconciliation is a widely-used technique for studying the evolutionary history of pairs of entities such as hosts and parasites, pairs of symbionts, and species and genes. In the duplication-transfer-loss (DTL) model, the biological events that are used to explain the possible discordance between pairs of tree are speciation, duplication, transfer, and loss.

Typically, reconciliation is performed using a maximum parsimony formulation. Maximum parsimony has been shown to accurately reconstruct simulated data where ground truth is known [1]. While alternative statistical approaches have also been explored, they have many more parameters that must be estimated and the algorithms are generally prohibitively slow [2, 3]. Nonetheless, it must be noted that all reconciliation methods are inherently limited by the evolutionary processes that they model. Moreover, while parsimony methods are appropriate for relatively simple evolutionary histories, they are likely to be less accurate for complex ones.

In the maximum parsimony framework, each type of event has an associated cost and the objective is to find a mapping of one tree (e.g., the gene tree) onto the other tree (e.g., the species tree) that minimizes the total cost of the events induced by that mapping. The maximum parsimony reconciliation problem in the DTL model has received considerable attention over the last decade due to its broad applicability. Efficient algorithms have been developed for the reconciliation problem [1, 4, 5] and have been implemented in a number of popular software tools [1, 4, 6, 7]. Hundreds of published studies in the life sciences have used these tools in their analyses.

Unfortunately, the number of maximum parsimony reconciliations (MPRs) can grow exponentially in the size of the trees [8]. Moreover MPRs often differ substantially from one another [9, 10]. In such cases, making inferences from a single maximum parsimony reconciliation can lead to conclusions that are not supported, and may even be contradicted, by other maximum parsimony reconciliations.

A fundamental problem, therefore, is that of identifying a set of best representative reconciliations. Prior work has included efforts to sample MPRs uniformly at random [8] and to find a single *median* MPR [11]. Recent work has demonstrated that MPR space is, in general, too diverse to be represented by a single MPR [9, 12]. Algorithms have been developed to implicitly cluster MPR space using *k*-medoids and *k*-centers [13], but these algorithms have several limitations. First, the asymptotic running times of these algorithms are *O*(*n*^*k*+3^ log*k*) where *n* is the size of the trees and *k* is the desired number of clusters. Thus, these algorithms are generally impractical except for very small datasets and numbers of clusters. Moreover, these clustering algorithms provide a representative reconcliation for each cluster but do not provide the clustering itself. Thus, it is not possible to compute various statistics on the clusterings nor to determine to which cluster an MPR belongs.

In this paper, we describe an efficient and practical method for clustering the space of MPRs using agglomerative hierarchical clustering. The hierarchical clustering method described here has a number of important properties. First, it is applicable to a variety of different objectives and linkage criteria. Second, the clusters are compactly represented as reconciliation graphs [14], which permits efficient algorithms to compute statistics on these clusters and to find one or more representative reconciliations in each cluster including *median reconciliations* [11] and *maximum event support reconciliations* [12], among others. Third, the asymptotic worst-case running time is practical for large trees, large values of *k*, and is not dependent on the number of MPRs. We demonstrate the viability of this approach on a large Tree of Life dataset [15] in which some trees induce more than 10^12^ MPRs.

In summary, the contributions of this paper are:

1. A general framework for agglomerative hierarchical clustering of MPR space;
2. Application of this method for two specific linkage criteria, one seeking to maximize the average event support in each cluster and the other seeking to minimize the distance between MPRs in each cluster;
3. Experimental results on a large biological dataset that demonstrate the viability and utility of this approach.

We provide an easily-extensible Python tool, called *cluMPR* (https://www.cs.hmc.edu/~hadas/clumpr), that implements this clustering method.

The next several subsections provide definitions that will be used to describe our algorithm. For consistency of notation and definitions, this material is taken directly from [9, 10] with permission.

### Maximum parsimony reconciliations

An instance of the DTL-MPR problem is a 6-tuple (*S*,*G*,*ϕ*,*d*,*t*,*ℓ*) where *S*=(*V*_*S*_,*E*_*S*_) and *G*=(*V*_*G*_,*E*_*G*_) are binary trees, *ϕ* is a function that maps the leaves of *G* to the leaves of *S*. This function need not be one-to-one nor onto. Parameters *d*, *t*, and *ℓ* are non-negative event costs for duplication, transfer, and loss events, respectively. These events are explained in detail below. The trees *S* and *G* are assumed to be undated, but all results in this paper can be easily adapted to dated trees as well.

A *reconciliation mapping* for a given instance is a function *Φ* that maps the vertices of *G* to the vertices of *S* such that *Φ*(*g*)=*ϕ*(*g*) for each leaf *g* of *G* and, if *g* is an internal vertex of *G* with children *g*^′^ and *g*^′′^, then (1) *Φ*(*g*) cannot be a descendant of either *Φ*(*g*^′^) or *Φ*(*g*^′′^) and (2) at least one of *Φ*(*g*^′^) or *Φ*(*g*^′′^) is equal to or a descendant of *Φ*(*g*).

A reconciliation mapping induces four types of events. Each internal vertex *g*∈*V*_*G*_ induces one speciation, duplication, or transfer event. In addition, an internal vertex may induce zero or more loss events. For an internal gene tree vertex *g*, with children *g*^′^ and *g*^′′^, the events induced by *Φ* are as follows:

- **Speciation event:** Vertex *g* induces a speciation event if one of *Φ*(*g*^′^) and *Φ*(*g*^′′^) is in the left subtree and the other is in the right subtree of *Φ*(*g*).
- **Duplication event:** Vertex *g* induces a duplication event if each of *Φ*(*g*^′^) and *Φ*(*g*^′′^) is either equal to or a descendant of *Φ*(*g*) but does not satisfy the requirements for a speciation event.
- **Transfer event:** Vertex *g* induces a transfer event if exactly one of *Φ*(*g*^′^) and *Φ*(*g*^′′^) is either equal to or a descendant of *Φ*(*g*) and the other is neither an ancestor nor a descendant of *Φ*(*g*).
- **Loss events:** Each non-root vertex *g* (including leaf vertices) may induce zero or more loss events as follows: Let *p*(*g*) denote the parent of *g* in tree *G*. If *Φ*(*p*(*g*)) is ancestral to *Φ*(*g*), then each species vertex *s* on the path from *Φ*(*p*(*g*)) to *Φ*(*g*) induces a loss event, except for *Φ*(*g*) and also not *Φ*(*p*(*g*)) if *p*(*g*) induces a speciation event. For each loss induced by a vertex *s* on the path from *Φ*(*p*(*g*)) to *Φ*(*g*), we say that *gpasses* through *s*.

**Fig. 1:**
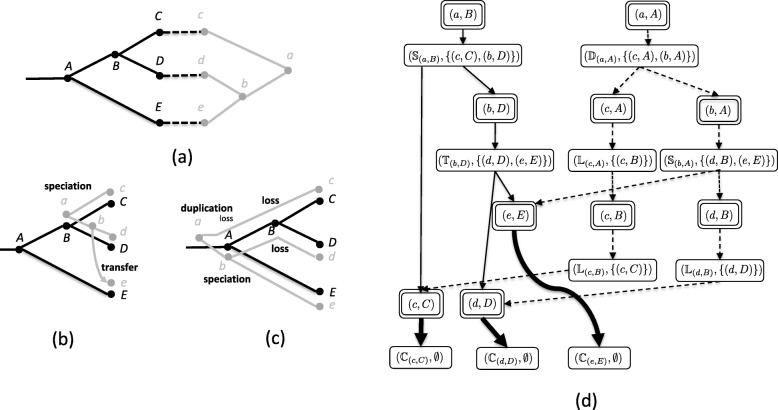
DTL reconciliation. **a** An instance of the DTL reconciliation problem comprising a species tree (black), a gene tree (gray), and a leaf mapping. Duplication, transfer and loss costs are 1, 4, and 1, respectively. **b** and **c** Two different MPRs, each with total cost 4. **d** The associated reconciliation graph. Mapping nodes are indicated with double line borders. Event nodes are designated with $\mathbb {S}$ (speciation event), $\mathbb {D}$ (duplication event), $\mathbb {T}$ (transfer event), or $\mathbb {L}$ (loss event). The reconciliation traversal indicated by solid edges corresponds to the MPR in (**b**) and the reconciliation traversal indicated by dashed edges corresponds to the MPR in (**c**); bold edges indicate shared elements of the two MPRs. Figure adapted from Haack et. al [9] with permission

The cost of a reconciliation mapping is defined to be the sum of the costs of all of the induced events. Typically, speciations events are considered null events and thus have cost zero. A minimum cost reconciliation mapping is called a *maximum parsimony reconciliation (MPR)*. Figure 1a shows an example of a DTL-MPR instance and Fig. 1b, c shows two different MPRs for that instance using duplication, transfer, and loss costs of 1, 4, and 1, respectively.

Using existing algorithms, a maximum parsimony reconciliation can be found in time *O*(|*G*||*S*|) [1, 2]. The problem becomes NP-complete, however, if the reconciliation is required to be temporally feasible which means that there exists a total ordering of the events such that an event involving a gene vertex *g* comes earlier in the ordering than any event involving a descendant of *g*. Fortunately, temporal infeasiblity can be detected when it occurs [2, 16].

### Reconciliation graphs and traversals

The space of all MPRs can be represented in polynomial space using a *reconciliation graph* (Fig. 1d). This representation was originally developed by Scornavacca et al. [14] for dated trees and later modified and adapted for undated trees [17]. For completeness, this representation is summarized below.

Consider a DTL-MPR instance (*S*,*G*,*ϕ*,*d*,*t*,*ℓ*). Let ***Φ*** denote the set of all MPRs for this instance. For a gene vertex *g*, let the children of *g* be denoted by *g*^′^ and *g*^′′^. Then, **events**(*g*,*s*) is the set of the following tuples induced by each MPR *Φ*∈***Φ***:

- $(\mathbb {S}_{(g,s)}, \{(g^{\prime }, s^{\prime }), (g^{\prime \prime }, s^{\prime \prime })\})$ for each speciation in which *g* is mapped to *s*, *g*^′^ is mapped to *s*^′^ or one of its descendants, and *g*^′′^ is mapped to *s*^′′^ or one of its descendants, where *s*^′^ and *s*^′′^ denote the children of *s*;
- $(\mathbb {D}_{(g,s)}, \{(g^{\prime }, s), (g^{\prime \prime }, s)\})$ for each duplication in which *g* is mapped to *s*.
- $(\mathbb {T}_{(g,s)}, \{(g^{\prime }, s), (g^{\prime \prime }, \hat {s})\})$ for each transfer in which *g* is mapped to *s* and one child, wlog *g*^′′^, is mapped to a vertex $\hat {s}$ that is not ancestrally related to *s*;
- $(\mathbb {L}_{(g,s)}, \{(g, s^{\prime })\})$ for each loss in which *g* passes through *s*, and *s*^′^ is the vertex that follows *s* on the path from *Φ*(*p*(*g*)) to *Φ*(*g*); and
- $(\mathbb {C}_{(g,s)}, \varnothing)$ for a contemporaneous leaf association where *g* and *s* are leaves and *ϕ*(*g*)=*s*.

Next, we make several observations about this representation. First, if *g* is mapped to *s* as a speciation event, the children of *g*, denoted *g*^′^ and *g*^′′^, are mapped to descendents of *s*. However, the speciation event is represented by associating *g*^′^ with one child of *s* (denoted *s*^′^) and associating *g*^′′^ with the other child of *s* (denoted *s*^′′^). Loss events are introduced for each loss incurred as *g*^′^ (or *g*^′′^) passes through species vertices on the path from *s*^′^ (or *s*^′′^) to *Φ*(*g*^′^) (or *Φ*(*g*^′′^)). Similarly, for a duplication event in which *g* is mapped to *s*, the children of *g* may be mapped to *s* or descendants of *s*. However, the duplication event is represented by associating both *g*^′^ and *g*^′′^ with *s* and then loss events are introduced for each loss on the path from *s* to *Φ*(*g*^′^) and on the path from *s* to *Φ*(*g*^′′^). Finally, if *g* is mapped to *s* as a transfer event, then one child of *g*, wlog *g*^′^, is mapped to *g* or one of its descendants while the other child, *g*^′′^ is mapped to a vertex $\hat {s}$ that is not ancestrally related to *s*. The transfer event is represented by associating *g*^′^ with *s* (and associating *g*^′′^ with $\hat {s}$); loss events are introduced for each loss on the path from *s* to *Φ*(*g*^′^).

For each such tuple *e*, let **type**(*e*) denote its first element, namely the event type and the ordered pair (*g*,*s*), and let **associations**(*e*) denote its second element, namely a set of zero or more ordered pairs. Note that if *e* corresponds to a speciation, duplication, or transfer event, then **associations**(*e*) is a set containing two ordered pairs, each representing an association between a gene tree vertex and a species tree vertex. If *e* is a loss event, then **associations**(*e*) is a set containing one such ordered pair indicating where the loss is incurred.

#### Reconciliation graph

The reconciliation graph contains a *mapping node* for each (*g*,*s*) pair where *g* is mapped to *s* in some MPR and, if not already included, a node (*g*,*s*) is also introduced if *g* passes through *s* due to a loss event. The reconciliation graph also contains an *event node* corresponding to each tuple in **events**(*g*,*s*). There is a directed edge from each mapping node (*g*,*s*) to each event node in **events**(*g*,*s*) and a directed edge from each event node *e* to a mapping node corresponding to an ordered pair in **associations**(*e*). (Throughout this paper, we use the term *vertex* for an element of the gene or species tree and the term *node* for an element of the reconciliation graph.)

The representation is compact by merit of the fact that, while a mapping (*g*,*s*) and its events may arise in many different MPRs, they are shared in this graph representation. Therefore, the size of the reconciliation graph is easily seen to be polynomial in the size of the two trees.

Ma et al. give a formal description of the algorithm for constructing undated reconciliation graphs, a derivation of its *O*(|*G*||*S*|^2^) running, and show that undated reconciliation graphs are acyclic [17]. Figure 1d shows the reconciliation graph for the DTL-MPR instance in Fig. 1a when duplication and loss have cost one and transfer has cost four.

#### Reconciliation traversal

Next, we define reconciliation traversals, which correspond to MPRs. Let $\mathbf {sources}(\mathcal {R})$ denote the set of source nodes of reconcilation graph $\mathcal {R}$ which, by definition, are mapping nodes of the form (*rg*,·) where *rg* represents the root of tree *G*.

For a reconciliation graph $\mathcal {R}$, a *reconciliation traversal* (abbreviated as *traversal*) is a subgraph of $\mathcal {R}$ whose root is a mapping node in $\mathbf {sources}(\mathcal {R})$. Each non-leaf mapping node added to the traversal has exactly one of its event node children added to the traversal. Each event node added to the traversal has all of its mapping node children added to the traversal. Figure 1d shows two traversals corresponding to the two MPRs in Fig. 1b, c.

There is a straightforward bijection between the set of MPRs and the set of traversals in the reconciliation graph [17]. A traversal, in turn, can be represented as the set of event nodes that it comprises. Thus, we may represent an MPR as the set of event nodes in the corresponding traversal. For an MPR *R*, let *E*(*R*) denote the set of event nodes in that reconciliation.

A reconciliation graph represents the space of all MPRs for a given pair of trees *G* and *S* their leaf associations, and their DTL event costs. We will represent subsets of that space, corresponding to clusters, using subgraphs of the reconciliation graph. A *reconciliation subgraph* is a subgraph of the reconciliation graph comprising the union of one or more traversals. Thus, a reconciliation subgraph includes at least one source node of the reconciliation graph, all of the sink nodes of the reconciliation graph, and some subset of the mapping and event nodes.

## Methods

In this section, we describe a general method for hierarchical clustering of MPR space and then provide examples of two specific applications of this method, one that seeks clusterings that maximize the average event support of the MPRs in each cluster and the other that seeks to minimize the average distance between MPRs in each cluster with respect to a given distance metric on MPRs.

Typically, agglomerative clustering algorithms are initialized with each item (e.g., MPR) forming its own cluster. Subsequently, pairs of clusters are merged according to the particular linkage criterion until the desired number of clusters is obtained. Since the appropriate number of clusters is often difficult to ascertain a priori, the pairing may continue until all the items are in a single cluster. By recording the intermediate clusterings, an appropriate number of clusters can be selected according to one of many different criteria [18, 19].

However, the initialization step for agglomerative clustering is, in general, not viable for MPRs since the number of such reconciliations can grow exponentially with the sizes of the trees [20]. Therefore, our approach is to begin the agglomerative clustering process with a small number of clusters, where each MPR is represented in one of those clusters. In other words, in the interest of computational efficiency, rather than starting the clustering process with a very large number of singleton clusters, we begin the process with a much smaller number of larger clusters. These initial clusters are constructed from the reconciliation graph and are represented by reconciliation subgraphs. Subsequently, when two clusters are agglomerated, their reconciliation subgraphs are merged. The number of initial clusters in our agglomerative clustering algorithm is denoted *N*; in the next section we show experimentally that this approach is effective for small values of *N*. In other words, the shortcut that is used to start the clustering with a small number of large clusters is both efficacious and computationally viable.

In the remainder of this section we describe the method for initializing the clusters, describe two linkage criteria, show that these criteria can be computed in polynomial time, and describe a method for identifying the presence of clusters.

### Representing and initializing clusters

To generate the initial clustering, we begin by selecting a depth level *L* to descend in the reconciliation graph. The set of sources of the reconciliation graph is said to be the set of level 0 subtraversals. For each source node in that set, we consider all of its event node children. Each source node, a single child event node, and the event node’s children (which are, by definition, mapping nodes) forms a level 1 subtraversal. In general, given the set of all level *i* subtraversals, we construct the set of all level *i*+1 subtraversals as follows: For each level *i* subtraversal, consider the set of all of its mapping node leaves. For each such mapping node, select one event node child and that event node’s children (which are, again, mapping nodes) to form a level *i*+1 subtraversal. This process is repeated, each time constructing all subtraversals at a given level, until we reach the set of all level *L* subtraversals. For each level *L* subtraversal, we add all of the nodes reachable from its leaves to form a reconciliation subgraph. These reconciliation subgraphs form the set of initial clusters. Note that this process has the desirable property that at the largest possible level, the subtraversals become complete traversals and we construct an initial clustering in which each cluster is a single MPR.

In our implementation of this algorithm, the user selects a desired number of initial clusters and the algorithm finds the smallest value of *L* that results in at least that many initial clusters. Let *N* denote the number of initial clusters actually found by this initialization step. Note that *N* may be larger than the desired number since the smallest level that generates at least the desired number of clusters depends on the reconciliation graph.

Henceforth, let *N* denote the number of initial clusters and let *n* and *m* denote the number of vertices in the species and gene trees, respectively.

#### **Lemma 1**

The number of nodes and edges in a reconciliation graph is bounded by *O*(*n*^2^*m*).

#### *Proof*

The number of mapping nodes is bounded by *O*(*nm*) since each mapping node associates a gene tree vertex with a species tree vertex. Each mapping node has a number of event node children bounded by *O*(*n*) since a mapping node may induce a speciation event in one of two ways, depending on which child of *g* is mapped to which child of *s*, it may induce a single duplication event, it may induce *O*(*n*) transfer events since one of the two children of *g* may be transferred to a different node of *S*, and may induce up to two loss event children depending on whether the loss occurs on the left or right child of *s*. Therefore, the total number of event nodes is bounded by *O*(*n*^2^*m*) and the total number of mapping and event nodes is bounded by *O*(*n*^2^*m*). Since each of the *O*(*nm*) mapping nodes has a number of children bounded by *O*(*n*) and each of the *O*(*n*^2^*m*) event nodes has at most two children, the number of edges is bounded by *O*(*n*^2^*m*). □

#### **Lemma 2**

The construction of the reconciliation subgraphs corresponding to the initial clusters takes time *O*(*Nn*^2^*m*).

#### *Proof*

The subtraversals can be constructed using breadth-first search starting from the sources of the reconciliation graph. By Lemma 1, the reconciliation graph has *O*(*n*^2^*m*) nodes and *O*(*n*^2^*m*) edges. Therefore, this process takes time *O*(*n*^2^*m*). Next, each of the *N* subtraversals is expanded into a subgraph of the reconciliation graph corresponding to an initial cluster, which takes time *O*(*Nn*^2^*m*). □

In the next two sections, we discuss linkage criteria for merging the initial clusters.

### Criterion 1: minimizing average distance

In this section we seek to find a set of clusters that minimizes the average distance between MPRs within each cluster with respect to a given distance metric. Let *d*(*R*_1_,*R*_2_) be a distance metric for any pair of MPRs, *R*_1_ and *R*_2_. For example, in the *symmetric distance metric*, the distance is the number of events that are in exactly one of the two MPRs, that is |*E*(*R*_1_)⊕*E*(*R*_2_)| where *E*(*R*) denotes the set of events in reconciliation *R* and ⊕ is the symmetric set difference operator [11]. In the *path distance metric*, the distance is defined as the sum, over all gene nodes *g*, of the length of the path from *s*_1_ to *s*_2_, where *g* is mapped to *s*_1_ in *R*_1_ and *g* is mapped to *s*_2_ in *R*_2_ [12, 21]. A number of other distance metrics for MPRs have been proposed as well [21, 22]. For concreteness, we use the symmetric distance metric here, although these results are applicable to other distance metrics as well.

Let ${\mathcal C} = \{C_{1}, C_{2}, \dots, C_{k} \}$ denote a *k*-clustering of MPR space. Let |*C*_*i*_| denote the number of MPRs in cluster *C*_*i*_ and let *μ*_*i*_ denote the average distance between all pairs of MPRs in *C*_*i*_ with respect to the given distance metric. The *weighted average distance (WAD)* of ${\mathcal C}$ is defined to be

$${WAD}({\mathcal C}) = \frac{\sum_{i=1}^{k} |C_{i}| \mu_{i}}{\sum_{i=1}^{k} |C_{i}|} $$

To optimize this objective function, a natural linkage criterion is to agglomerate the pair of clusters that gives the largest reduction in the weighted average distance, which is effectively a gradient descent heuristic on this objective function. The computation of the average distances between MPRs in a reconcilation graph can be performed in polynomial time [10] in spite of the fact that the number of MPRs may be exponentially large.

#### **Lemma 3**

The running time of the clustering algorithm for weighted average distance is *O*(*N*^2^*n*^4^*m*^2^ log*m*).

#### *Proof*

Computing the number of MPRs in the reconciliation subgraph can be performed in time *O*(*nm*) [1] and computing the average distance between all pairs of MPRs can be performed in time *O*(*n*^4^*m*^2^ log*m*) [10].

By Lemma 2, construction of the initial clustering can be performed in time *O*(*Nn*^2^*m*). We then compute the average distance and number of MPRs for each of the *N* initial clusters in time *O*(*Nn*^4^*m*^2^ log*m*).

Next, we compute and record the weighted average distance when merging each pair of initial clusters. This requires *O*(*N*^2^) computations of the average distance and number of MPRs, for a total of *O*(*N*^2^*n*^4^*m*^2^ log*m*) time.

On each of the *O*(*N*) successive iterations, it takes *O*(*N*) time to identify the pair of clusters to merge. Computing the average distance and number of MPRs in that cluster takes time *O*(*n*^4^*m*^2^ log*m*) and merging the two reconciliation subgraphs takes time *O*(*n*^2^*m*) since, by Lemma 1, each of the two subgraphs being merged has *O*(*n*^2^*m*) nodes and edges. Finally, we must compute the distance and number of MPRs between the new (merged) graph and the other *O*(*N*) graphs, which requires *O*(*N*) computations of the average distance and number of MPRs. Thus, each merging iteration requires *O*(*Nn*^4^*m*^2^ log*m*) time, and the *O*(*N*) iterations take *O*(*N*^2^*n*^4^*m*^2^ log*m*) time in total. The total running time of the clustering algorithm is, therefore, bounded by *O*(*N*^2^*n*^4^*m*^2^ log*m*). □

### Criterion 2: maximizing average event support

Another objective of interest is to find a clustering that maximizes the average event support in each cluster. For each event found in an MPR in a given cluster, the support (or *frequency*) for that event is the fraction of MPRs in that cluster that include that event [11]. In many cases, a significant fraction of events have very low support over the space of all MPRs [12]. Thus, it may be desirable to partition MPR space into clusters, where the average event support with respect to the MPRs in each cluster is higher than in the entire space of MPRs. By selecting a representative MPR in each cluster, we can again obtain a set of MPRs that better represent the diversity of MPR space than could be done by selecting a single MPR drawn from the entire space.

Let ${\mathcal C} = \{C_{1}, C_{2}, \dots, C_{k} \}$ denote a *k*-clustering of MPR space. Let *σ*_*i*_ denote the average event support in cluster *C*_*i*_. The *weighted average support (WAS)* of ${\mathcal C}$ is defined to be

$${WAS}({\mathcal C})= \frac{\sum_{i=1}^{k} |C_{i}| \sigma_{i}}{\sum_{i=1}^{k} |C_{i}|} $$

To optimize this objective function, a natural linkage criterion selects the pair of clusters whose agglomeration gives the largest increase in the weighted average support, which is a gradient ascent heuristic on this objective function. The computation of event frequencies can be computed in polynomial time [11].

#### **Lemma 4**

The running time of the clustering algorithm for weighted average support is *O*(*N*^2^*n*^2^*m*).

#### *Proof*

The analysis is identical to that in Lemma 3 except that the computation of average distance is replaced by the computation of support values, which can be computed in time *O*(*n*^2^*m*) [12]. Thus, the initial construction of the clustering takes time *O*(*Nn*^2^*m*) and the subsequent clustering takes time *O*(*N*^2^*n*^2^*m*). Thus, the total running time is bounded by *O*(*N*^2^*n*^2^*m*). □

### The improvement score

To analyze the performance of the hierarchical clustering method, we define an *improvement score*. For the average event support criterion, which is a maximization problem, the improvement score for a given clustering is the weighted average support for the clustering divided by the weighted average support for the entire MPR space, which is simply the average event support. The improvement score indicates the improvement in intracluster support values using clustering versus using no clustering. For the average distance criterion, which is a minimization problem, we invert this ratio: The improvement score for a given clustering is the average pairwise distance between all MPRs divided by the weighted average distance for the clustering. In this case, the improvement score indicates the improvement in the intracluster distances using clustering versus using no clustering.

Note that the improvement score compares a clustering of size *k* to no clustering or, equivalently, a clustering of size *k* to a clustering of size 1. A related measure of interest is the improvement achieved by going from *k*−1 clusters to *k* clusters, for *k*≥2. Let *C*_*a*_ and *C*_*b*_ be the clusters agglomerated by the algorithm when *k* clusters are reduced to *k*−1 clusters or, equivalently, when *k*−1 clusters are split into *k* clusters. Let *C*_*ab*_ denote the agglomeration of those two clusters. The *local improvement at k*, *k*≥2, denoted *WASlocal*_*k*_ for weighted average support, is defined to be:

$${{WASlocal}_{k}} = \frac{{WAS}(\{C_{a}, C_{b}\})}{{WAS}(\{C_{ab}\})} $$

Similarly, for weighted average distance, the local improvement at *k* is denoted *WADlocal*_*k*_ and is defined to be:

$${{WADlocal}_{k}} = \frac{{WAD}(\{C_{ab}\})}{{WAD}(\{C_{a}, C_{b}\})} $$

Note that when the local improvement is relatively small (e.g., close to 1), there is little improvement in the objective function due to splitting *C*_*ab*_ into *C*_*a*_ and *C*_*b*_. Conversely, when this score is relatively large, the objective function improves due to the splitting. Therefore, by identifying the value(s) of *k* where the local improvement score is relatively large, we can identify potentially appropriate number(s) of clusters.

## Results

We applied our algorithm to a widely-used Tree of Life dataset comprising 100 primarily prokaryotic species and 4849 gene trees [15] using duplication, transfer, and loss costs of 2,3,1, respectively [15, 23]. While these costs have been used in many studies, the xScape algorithms and tools provide a systematic approach for selecting event costs for a given dataset and we recommend using those in practice [24]. We randomly sampled 100 of the 4849 gene trees that induced at least 1000 MPRs since the clustering problem is of particular interest in large MPR spaces. Some gene families in this set induced over 10^12^ MPRs. The 100 selected gene trees had between 20 and 299 leaves.

### Running times

We used a commodity server (AMD Opteron 6276 2.3 GHz, 503 GB RAM) for our experiments. We used *N*=25 for the minimum initial number of clusters since we found little benefit to using a larger value of *N* as discussed below. We set a 20-min timeout for each tree which resulted in some timeouts. Running times and the number of timeouts are summarized in Table 1.

**Table 1:** Running times for the 100 gene families that were clustered using weighted average support and distance linkage criteria

Objective	Mean runtime (s)	Standard deviation (s)	*#* Timeouts
Support	25.70	61.99	5
Distance	266.72	272.71	13

### Impact of the number of initial clusters

**Fig. 2:**
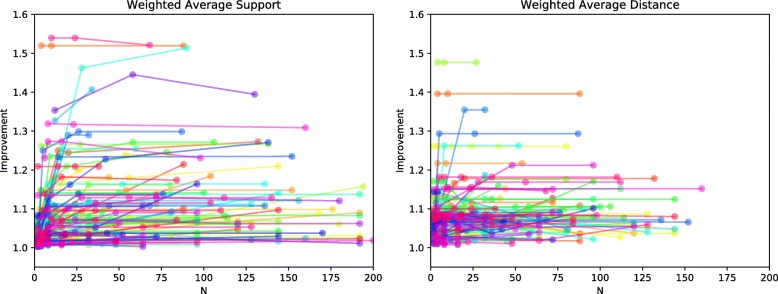
Improvement of two clusters versus no clustering as a function of the size of the initial clustering, *N*. Each color represents a different gene tree. The sizes of the initial clusterings varies among gene trees due to differences in their reconciliation graphs

**Fig. 3:**
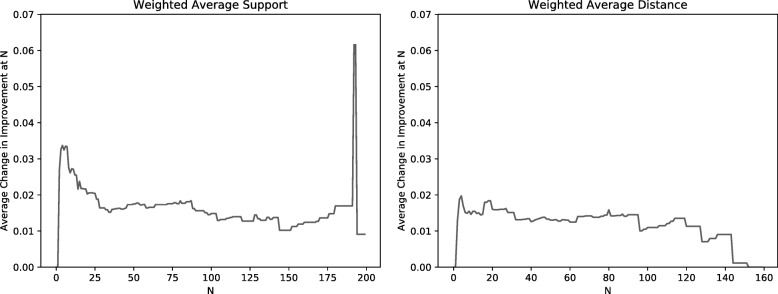
The average change in improvement from Fig. 2 as a function of the size of the initial clustering, *N*. For each value of *N*, each gene family that gives rise to an initial clustering of size less than *N* and one of size greater than *N* is considered. The improvement between those two clustering sizes is measured and averaged over all such gene families. For large values of *N*, the number of families that had a clustering of size less than *N* and also one of size greater than *N* but less than our maximum of 200 is very small. Thus, for values of *N* near 200 the number of samples is small and any statistic for those values is therefore susceptible to error

The efficiency of this clustering method depends on using a relatively small number of initial clusters, denoted by the parameter *N*. In theory, starting with a very large number of clusters (e.g., singleton clusters, each comprising a single MPR) should produce better final clusterings than starting with a small number of larger initial clusters since those initial clusters are simply constructed from the topology of the reconciliation graph and not iteratively by applying the linkage criterion beginning with clusters of size one. Thus, we investigated the relationship between the improvement score and the number of initial clusters. Specifically, we measured improvement as a function of *N* for *k*=2 because *k*=2 is the last iteration of the algorithm and thus incorporates the agglomeration choices from all previous iterations. The results are summarized in Fig. 2. Figure 3 shows the change in improvement as a function of *N*. Note that the sharp spike and drop-off at the right ends of the two plots are due to very small sample sizes for those values of *N* and thus should not be considered in this evaluation. The average change in improvement is very small across this range of *N*, indicating that the quality of the clusterings is not strongly dependent on the number of initial clusters.

### Improvement as a function of *k*

**Fig. 4:**
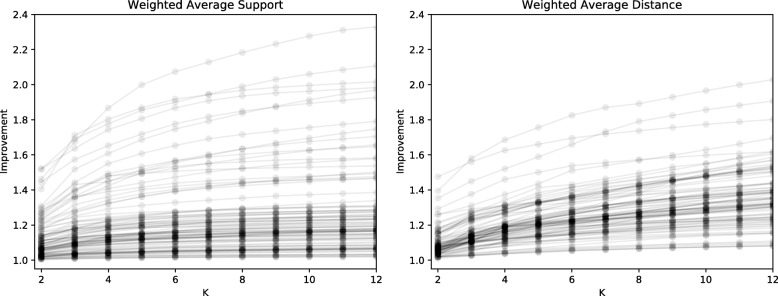
Improvement as a function of the number of clusters, *k*. Each curve represents a single gene tree. The improvement is relative to the score for a single cluster

**Fig. 5:**
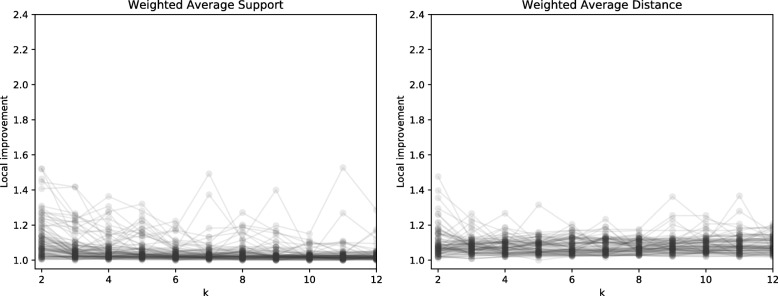
Local improvement as a function of the number of clusters, *k*. Each curve represents a single gene tree

We explored how improvement (the ratio between the objective function at *k* clusters versus 1 cluster) changes as a function of *k*. These results are summarized in Fig. 4. For some gene families, improvements were consistently close to 1.0, meaning that there is not evidence of clusters in their MPR spaces. However, cases in which the improvement score is relatively large suggest that clusters exist. Figure 5 shows local improvement of *k* (the improvement resulting from splitting *k*−1 clusters into *k* clusters). The values of *k* that are relatively large indicate candidates for an appropriate number of clusters. Collectively, these results indicate that while some gene families do not give rise to clusters, a number of gene families appear to have two clusters and some have an even larger number of clusters.

### Impact of the number of mPRs

**Fig. 6:**
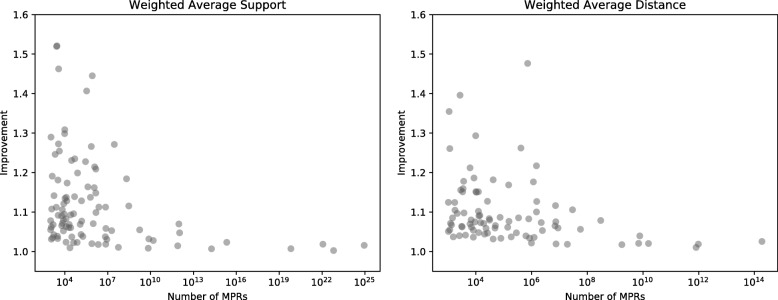
Number of MPRs in the original reconciliation graph versus improvement after forming two clusters

Figure 6 shows the relationship between the improvement score (from no clustering to two clusters) and the number of MPRs. We found that there is no correlation between improvement score and the number of MPRs, implying that the presence of clusters is not dependent on the size of MPR space for this dataset.

### Correlation between linkage criteria

**Fig. 7:**
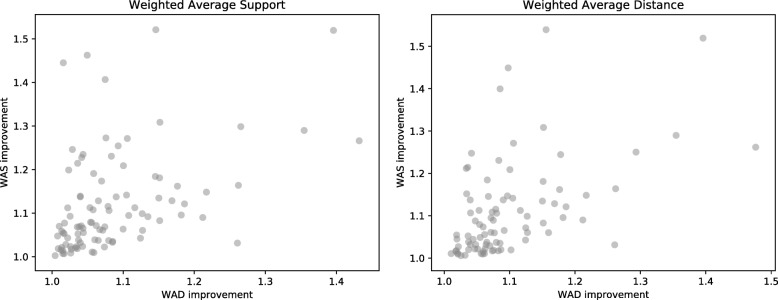
Relationship between the two linkage criteria for two clusters. On the left, clusters were generated for each family using the WAS objective, then evaluated using both. On the right, clusters were generated using the WAD objective

Different linkage criteria may result in different clusterings. Figure 7 summarizes the relationship between the clusterings using weighted average support and weighted average distance, using improvement scores to measure the strength of the relationship. The plot at left in Fig. 7 shows the results of finding two clusters using the average weighted support criterion and evaluating the improvement in both weighted average support and weighted average distance on the resulting clusters. The plot at right in Fig. 7 shows the analogous results when clustering using the average weighted distance criterion. There is a small but statistically significant positive correlation between the two improvement scores (*r*=0.43, *p*=9.38×10^−6^, *n*=95 for clusters obtained using event support, and *r*=0.51, *p*=3.87×10^−7^, *n*=85 for clusters obtained using pairwise distance). These results indicate that in some cases, clusters arise regardless of which of the two linkage criteria are used. However, in general, the two linkage criteria are sufficiently different that the presence of clusters under one criterion does not necessarily imply clusters using the other criterion. Further work is required to assess which linkage criteria are most meaningful and useful in practice.

## *cluMPR* software tool

A Python implementation of the agglomerative hierarchical clustering algorithm is available in the *cluMPR* tool (www.cs.hmc.edu/~hadas/clumpr). This tool supports clustering using the weighted average support and the weighted average distance linkage criteria, allows for a median reconciliation to be generated from each cluster as the representative of that cluster, and is extendible to other linkage criteria. The tool generates various types of analyses and plots such as those shown in the previous section.

We conclude with an example of how the cluMPR tool can be useful and how the results can be interpreted. For this example, we chose the gene tree (COG1230) from the 100 trees in our sample that gave the largest improvement for weighted average distance (1.48) for *k*=2. This tree induced 718848 MPRs.

**Fig. 8:**
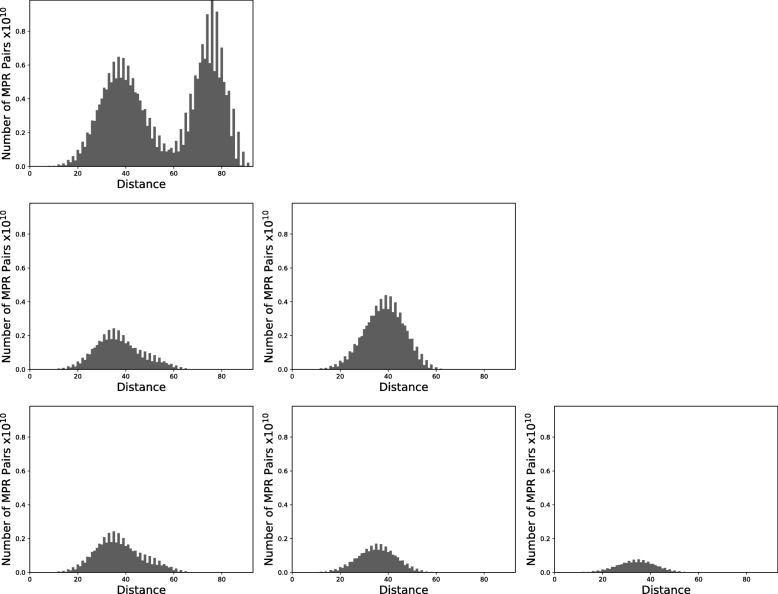
Results of clustering using the weighted average distance criteria for gene family COG1230 from the Tree of Life data set using DTL costs 2, 3, 1, respectively with *N*=27. On the top are the pairwise distances for the entire MPR space. The second row shows the pairwise distances for *k*=2 clusters. The third row shows the pairwise distances for *k*=3 clusters obtained using the same method. In this case, the initial distribution is bimodal, suggesting the presence of multiple clusters. For *k*=2, the local improvement is 1.48 and both distance distributions are unimodal, indicating that two clusters were identified. For *k*=3, the local improvement is 1.10 and the distributions are remain unimodal, suggesting the presence of just two clusters

We used the hierarchical clustering algorithm to cluster the MPR space using the weighted average distance linkage criterion. Figure 8 shows the distribution of distances between all MPRs at the top left. The second row shows the distribution of pairwise distances for two clusters (*k*=2) and the third row shows the pairwise distances for three clusters (*k*=3).

**Fig. 9:**
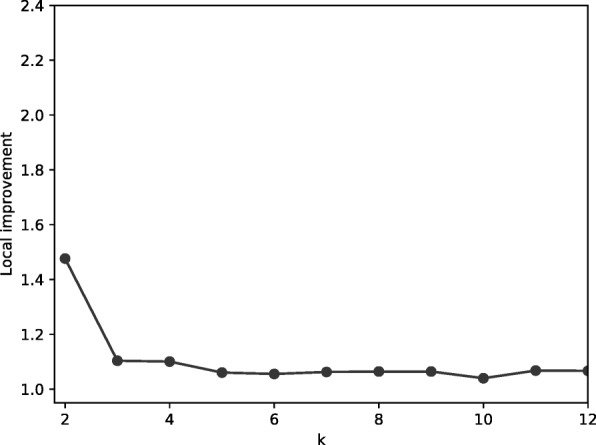
Local improvement for *k* using weighted average distance for gene family COG1230. The relatively high local improvement at *k*=2 and low local improvement for larger values of *k* suggests that there are two clusters in this space

There is strong evidence for two clusters in this example since the original bimodal distribution resolves into two clusters with unimodal distance distributions. However, the local improvement drops from 1.48 at *k*=2 to 1.10 at *k*=3 and the distributions remain unimodal at *k*=3. Moreover, as shown in Fig. 9, the local improvement remains relatively close to 1 for larger values of *k*, further supporting the hypothesis that there are not more than two clusters in this case.

## Conclusion and future work

In this paper we have described an agglomerative hierarchical clustering methodology for the space of maximum parsimony reconciliations in the duplication-transfer-loss model. We have demonstrated this method for two different linkage criteria and have shown that the worst-case asymptotic running time is polynomial in the sizes of the trees and the size of the initial clustering. Using the improvement score measure, we have shown experimentally that this method is effective even for small initial clusterings. Thus, this approach provides an efficient way to identify clusters in MPR space. From each cluster, we can then select one or more representative MPRs (e.g, median MPRs or maximum average event support MPRs). Therefore, we believe that this method provides a useful way to identify a best set of representative MPRs when MPR space is too diverse to be adequately represented by a single MPR.

A number of challenges remain for future work. First, determining the appropriate number of clusters in an MPR space remains an important problem. We have offered one approach using local improvement scores, but other techniques such as silhouettes [18] and gap statistics [19] are potentially applicable and merit investigation. Second, the relative merits of the two linkage criteria described here, as well as other possible criteria, also merit exploration and evaluation. Finally, while the Tree of Life dataset used here is large and diverse, experimental studies using other datasets and event costs are also of potential interest.

## Data Availability

The software in this paper and the data used in the experiments are available at https://www.cs.hmc.edu/~hadas/clumpr.

## Abbreviations

DTL: Duplication-transfer-loss
MPR: Maximum parsimony reconciliation
PDV: Pairwise distance vector
WAD: Weighted average distance
WAS: Weighted average support
